# Ultra-long-term results of the Chiari pelvic osteotomy in hip dysplasia patients: a minimum of thirty-five years follow-up

**DOI:** 10.1007/s00264-023-05912-9

**Published:** 2023-08-25

**Authors:** Catharina Chiari, Eleonora Schneider, Tanja Stamm, Philipp Peloschek, Rainer Kotz, Reinhard Windhager

**Affiliations:** 1 Division of Orthopaedics, Department of Orthopaedics and Trauma-Surgery, Medical University of Vienna, Waehringer Guertel 18-20, 1090 Vienna, Austria; 2 Center for Medical Statistics, Informatics and Intelligent Systems, Institute of Outcomes Research, Medical University of Vienna, Spitalgasse 23, 1090 Vienna, Austria; 3 Radiology Center, Lazarettgasse 25, 1090 Vienna, Austria; 4 Vienna Private Hospital, Pelikangasse 15, 1090 Vienna, Austria

**Keywords:** Chiari pelvic osteotomy, Long-term follow-up, Developmental dysplasia of the hip, Hip arthroplasty

## Abstract

**Purpose:**

The Chiari pelvic osteotomy was the first surgical procedure to address hip dysplasia by changing the position of the acetabulum by medialization, thus creating a bony roof and improving biomechanical conditions. The aim of this retrospective cohort study was to report on the very long-term results of this technique.

**Methods:**

Out of a consecutive series of 1536 hips, 504 in 405 patients were available for follow-up. The patients were assessed by physical and radiological examination. A Kaplan–Meier survival analysis with total hip arthroplasty as an endpoint was performed and stratified for age groups, pre-operative diagnosis, sex and osteoarthritis stage.

**Results:**

The average follow-up was 36 ± 8.1 years (range, 35.2 to 54). The average pain level on the Visual Analogue Scale was 2.9 ± 2.6 (range 0 to 8.7). The average Harris Hip Score was 80.2 ± 17.4 (range 17.4 to 100). Correction of dysplasia was effective and remained stable over time. Osteoarthritis significantly increased over time with 53% Tönnis grade 3 at follow-up. The cumulative survivorship was 79.8% (95% confidence interval (CI), 76.1–83.2%) at 20 years, 57.1% (95% CI, 52.8–61.8%) at 30 years and 35% (95% CI, 30.3–40.3%) at 40 years. Young age, male sex and low osteoarthritis grade were positive prognostic factors.

**Conclusions:**

Although the Chiari pelvic osteotomy is considered a salvage procedure nowadays, it achieved excellent long-term results even in indications, which would be treated differently today. Young patients without osteoarthritis had the best outcome.

## Introduction

**Fig. 1:**
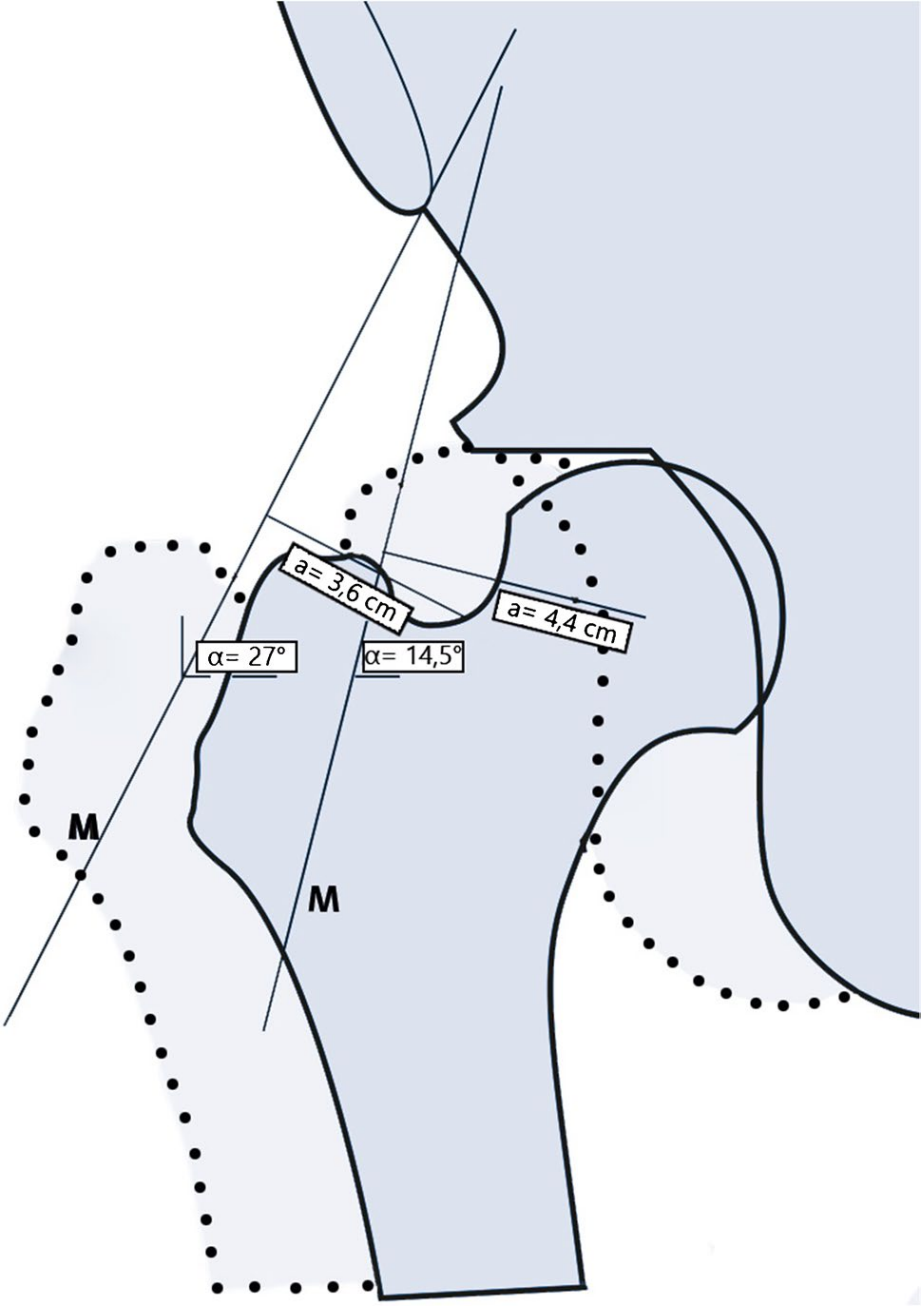
Medialization of the hip joint by the CPO leads to a lengthening of the lateral lever arm (a, a´) and a more vertical orientation of the pelvitrochanteric muscles (α,α´) with increasing abductor force (M, M´). At the same time, joint load is decreased

Various surgical techniques have been described for the treatment of acetabular deficiency in hip dysplasia [1–8]. Today, the Dega, Pemberton and Salter osteotomies are the most widely used in young children [3–5], while different types of triple osteotomies are recommended in older children with an open triradiate cartilage [9, 10]. In the skeletally mature pelvis, the periacetabular osteotomy (PAO) developed by Ganz is the gold standard for reorientation of the acetabulum [7], also the Tönnis triple osteotomy is used by a number of surgeons [6]. Mid- and long-term results show that those methods are safe and clinically successful [11–13]. The Chiari pelvic osteotomy (CPO) was the first surgical procedure to address the problem of hip dysplasia by splitting the iliac bone completely and changing the position of the acetabulum by medialization of the hip joint thus creating a bony roof and improving biomechanics (Fig. 1 Changes in Biomechanics of the Hip Joint after CPO) [2]. The new bony roof is not covered by articular cartilage. Instead, the interposed capsule and labrum are transformed into fibrocartilage with time. Therefore, osteoarthritis (OA) is expected to develop rather early. These disadvantages have narrowed the indication to a salvage procedure for severe dysplasia with lateralization of the femoral head and incongruent joints. However, mid- and long-term results from our institution were very good [14, 15] and we are now able to present the final follow-up of a unique cohort of patients operated by Chiari himself or his direct pupils between 1953 and 1986. This cohort comprises age groups and surgical indications that we treat differently nowadays. Therefore, we expected important information about the very long-term effectiveness and limitations of this surgical technique, which can be considered a historical milestone of orthopaedic surgery.

## Material and methods

### Patients

**Fig. 2:**
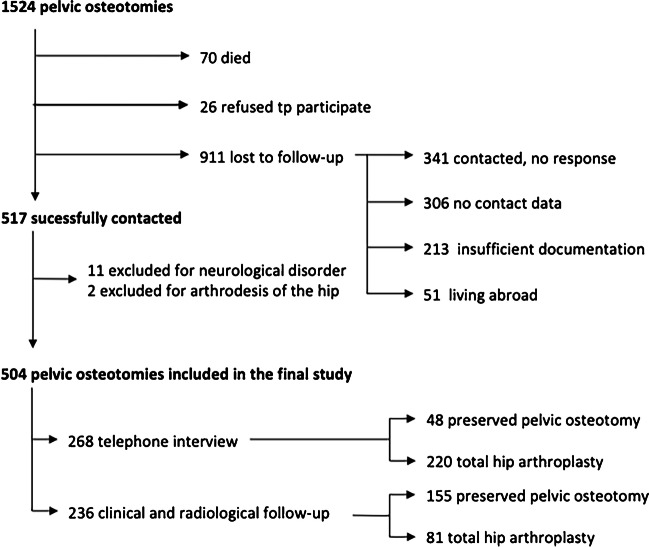
The flowchart shows selection and eligibility for the long-term follow-up study of hips treated with CPO

**Fig. 3:**
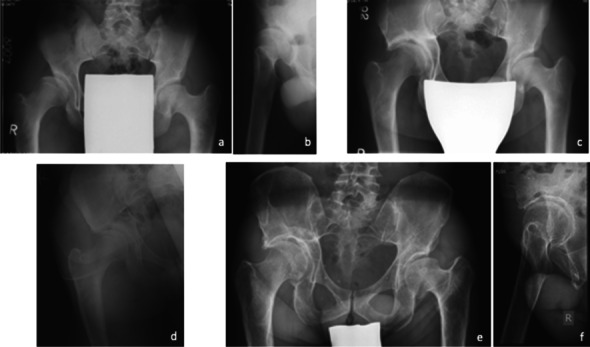
Male patient, CPO at age of 17 years with severe hip dysplasia. The right hip shows a L-CEA of 0 and a A-CEA angle of 14°, OA grade 0 (**a**, **b**). After CPO the acetabulum was medicalized by 47.6% and full coverage of the femoral head was achieved (**c**). The three year post op X-ray shows remodeling of the acetabulum and a congruent joint (**d**). After thirty years the patient had full ROM, negative Trendelenburg sign and pain of 2.2 out of 10 on the VAS. The L-CEA was 54 and the A-CEA 64°, OA grade 2 (**e**, **f**)

A data analysis of patients treated with CPO was performed. The study was approved by the institutional review board. The authors affirm that participants provided informed consent for publication of the images in Fig. 3a–f. We screened patient charts and radiographs from the time period between 1953 and 1986. A total of 1536 hips with CPO for hip dysplasia were identified. Patients were contacted by mail or telephone and invited to a follow-up visit. Finally, the study cohort consisted of 405 patients (355 women, 50 men) with 504 affected hips (260 left, 244 right, 99 of those bilateral) (Fig. 2 Overview of Study Cohort).

### Surgical technique

All patients were operated according to the original method developed by Karl Chiari [2]. The detailed technique has been described previously [15]. Originally, there was no internal transfixation of the osteotomy, but a hip spica-cast in abduction was applied for 3 to 4 weeks, since 1982, two parallel 2-mm Kirschner wires were used.

### Clinical evaluation

Informed consent was obtained from each participant. Patient demographics, pre-operative diagnosis, previous conservative treatment and previous or concomitant hip surgeries were documented (Table 1). Patient satisfaction was categorized, the modified Harris Hip Score (HHS) [16] was used to evaluate hip function and pain, a horizontal 10-cm Visual Analogue Scale (VAS) for pain assessment and the Short-Form (SF)-36 for overall health assessment [17]. Physical examination included range of motion, anterior impingement testing, leg length discrepancy, Trendelenburg sign and gait.

**Table 1:** Patients’ characteristics*

Variable	Summary statistics
Age at surgery**	23 ± 13 (2 to 55)
Male/female***	50 (12.3%) 355 (87.7%)
Total number of hips/right hips/left hips/bilateral***	504 (100%)/244 (48.4%)/260 (51.6%)/99 (19.6%)
BMI at follow-up (kg/m^2^)**	25.5 ± 4.3 (15.2 to 43.8)
Previous ipsilateral closed reduction and cast	319 (63.3%)
Previous ipsilateral hip surgery***	121 (24%)
Open reduction of the dislocated hip	37 (31%)
Derotation osteotomy of the femur	26 (21%)
Intertrochanteric varus/valgus osteotomy of the femur	39 (32%)
Acetabuloplasty	17 (14%)
Advancement of the greater trochanter	2 (2%)
Indication for surgery (preoperative diagnosis)***	
Congenital hip dislocation	32 (6.3%)
Hip dysplasia	287 (56.9%)
Hip dysplasia with subluxed femoral head	64 (12.7%)
Dysplastic osteoarthritis	109 (21.6%)
Not documented	12 (2.4%)

*Study cohort of 504 hips. **The values are presented as the mean and the standard deviation with the range in parentheses. ***The values are presented as the number, with the percentage in parentheses

### Radiological evaluation

We evaluated pre-operative, post-operative and follow-up X-rays. At follow-up, an ap pelvis, an axial view and a false profile view of both hips were obtained. Radiographs were measured digitally in the PACS System (Impax, Agfa Health Care). We measured the lateral centre-edge angle (L-CEA) of Wiberg [18], the anterior centre-edge angle (A-CEA) of Lequesne and de Seze [19], the acetabular head index (AHI), the acetabular index depth to width (AI) of Heyman and Herndorn [20] and the ACM angle of Idelberger and Frank [21]. Stage of OA was graded according to Tönnis [22, 23]. We evaluated joint congruency and femoral head shape on the final follow-up X-rays. Orientation of the osteotomy was judged as follows: ideal (10–15° ascending), too flat (< 10°), too steep (>15°), descending (negative angle), too high (> 1 cm above the acetabular rim) or intraarticular (entering the acetabulum). Medialization of the distal fragment was expressed as the percentage of the width of iliac wing.

### Statistical analysis

Descriptive statistics including absolute and relative frequencies as well as measures of central tendency and variation were calculated depending on the scale level and the distribution of the variables. Differences between subgroups in continuous, not normally distributed variables were tested with Mann–Whitney–Wilcoxon tests. Differences between categorical variables were assessed using chi square tests. In non-normally distributed variables, differences between repeatedly measured radiological angles at the time points pre-op, post-op and follow-up were calculated using Friedmann tests. In case of significant results, pairwise Mann–Whitney–Wilcoxon tests between each of the two time points were assessed. The Bonferroni method was used to control for multiple testing.

Kaplan–Meier estimates [24] for survival of the CPO with total hip arthroplasty (THA) as an endpoint were fitted for all patients together as well as stratified for sex, age group (group 1: <11 years *n* = 101, group 2: ≥ 11 years to 16 years *n* = 99, group 3: ≥ 16 years to 45 years *n* = 289, group 4 > 45 years *n* = 15), pre-operative diagnosis (group 1: hip dysplasia, group 2: dysplastic subluxation, group 3 congenital hip dislocation, group 4: OA), pre-operative conservative or surgical treatment and stage of OA. Potential statistically significant differences were assessed with log-rank test between the strata.

### Source of funding

This study was supported by the Research Grant of the Association for Orthopaedic Research (AFOR).

## Results

### Clinical results

The average follow-up was 36 ± 8.1 years (range, 35.2 to 54). A total of 203 hips had the preserved status post CPO and 301 had been replaced by a THA. The average age at surgery was 23 ± 13 years (range, 2 to 55). The average age at follow-up was 59.0 ± 11.9 (range, 31 to 87.1) for the whole cohort, 54.2 ± 10.7 (range, 31 to 83.4) for the patients with preserved hip joints and 62.2 ± 11.6 (range, 38.1 to 87.1) for the patients with THA, respectively. The body mass index (BMI) at follow-up was 25.5 ± 4.3 kg/m^2^ (range, 15.2 to 43.8). Previous or concomitant treatments as well as indications are listed in Table 1. Data for patients’ satisfaction was available for 484 of the 504 hips: in 359 (76%), it was very good, in 64 (13.6%) good, in 27 (5.7%) satisfactory, in 25 (5.2%) sufficient and in nine (1.9%) unsatisfactory. For the patients with the preserved hips, the results were as follows: the average pain level on the VAS was 2.9 ± 2.6 (range 0 to 8.7). The average HHS was 80.2 ± 17.4 (range 17.4 to 100). The functional subscale was 39.3 ± 9.2 (range 7 to 47 points) and the pain subscale 34.7 ± 10.1 (range 10 to 44), respectively. The deformity subscale of the 155 patients, who had been physically examined, showed a mean of 3.6 ± 1.3 (range 0 to 4). Average range of motion was 87.3° ± 20.7° (range 10 to 130) of flexion, 37.1° ± 12.7° (range 0 to70) of abduction, 28.0° ± 11.1 (range 0 to 50) of adduction, 23.0° ± 14.2° (range 0 to 80) of external rotation and 14.6° ± 12.1° (range 0 to 60) of internal rotation. The anterior impingement test was positive in 23 hips (14.7%). A positive Trendelenburg sign was found in 44 hips (28.4%), and a limp was present in 71 hips (45.8%). Leg length was unequal in 87 cases (56.1%); in 57 of those, the operated side was 1.0 ± 1.6 cm (range 0 to 6) shorter and in 30, it was 0.4 ± 0.8 cm (range 0 to 4) longer. The SF-36 patients’ health status was below the average values of a normative population concerning the physical components, whereas the average scores of the mental components were comparable to those of a normative population [25] (Table 2).

**Table 2:** Results of the SF-36

Subscales	overall	Sex		Age in years				
		Male	Female	30–39	40–49	50–59	60–69	70–79
Physical functioning	63.4 ± 26.4 (0–100)	72.6±24.5 (15–100)	61.3±26.5 (0–100)	73.4±17.1 (50–95)	73.7±22.3 (25–100)	59.4±27.0 (5–100)	52.3±28.5 (0–100)	61.7±30.6 (35–95)
Physical role functioning	69.7 ± 40.4 (0–100)	83.8±27.2 (25–100)	66.7±42.2 (0–100)	89.3±19.7 (50–100)	76.5±36.4 (0–100)	71.0±41.0 (0–100)	51.1±44.0 (0–100)	66.7±57.7 (0–100)
Bodily pain	54.7 ± 28.4 (0–100)	59.7±22.1 (32–100)	53.6±29.6 (0–100)	69.3±21.9 (41–100)	63.6±29.5 (0–100)	50.7±28.9 (0–100)	45.1±24.8 (0–100)	50.7±9.5 (41–60)
General health perception	66.9 ± 19.7 (22–100)	67.3±19.9 (22–100)	66.8±19.8 (25–100)	67.7±17.2 (47–92)	71.8±17.6 (30–100)	68.5±21.2 (25–97)	56.9±18.6 (22–82)	55.7±13.7 (40–65)
Vitality	60.7 ± 17.6 (0–100)	66.7±16.5 (20–85)	59.4±17.6 (10–95)	61.4±20.6 (30–85)	65.3±12.6 (30–85)	59.7±18.1 (10–95)	54.3±21.1 (20–85)	68.3±20.2 (45–80)
Social functioning	84.8 ± 21.0 (12.5–100)	95.2±10.1 (63–100)	82.4±22.1 (13–100)	85.7±23.3 (38–100)	87.1±18.1 (38–100)	84.6±23.0 (13–100)	79.0±21.3 (38–100)	100±0 (100–100)
Emotional role functioning	83.8 ± 35.0 (0–100)	98.3±7.7 (67–100)	80.7±37.7 (0–100)	83.3±40.8 (0–100)	92.2±21.8 (0–100)	84.1±35.6 (0–100)	71.7±44.9 (0–100)	66.7±57.7 (0–100)
Mental health	75.3 ± 17.5 (20–100)	77.8±14.6 (36–96)	74.7±18.1 (20–100)	62.8±25.4 (20–88)	77.2±14.7 (36–96)	74.2±18.4 (32–100)	77.8±15.7 48–100)	76±28 (44–96)

Results SF 36 of 115 patients (141 hips, average age 52.6 ± 8.7 (range, 31 to 71)). Data of 10 patients (14 hips) is missing. Results in mean±SD (range)

### Radiographic results

The L-CEA, the AHI, the AI and the ACM were significantly improved by the osteotomy. The lateral coverage tended to be above normal values reflected by high L-CEAs and a very low AHI. The average AHI and ACM values were within the normal or slightly pathological range. The angles remained stable from the post-operative to the follow-up X-ray. Only few pre- and post-operative false profile X-rays were available. At follow-up, the average A-CEA was corresponding to sufficient acetabular coverage. The medialization of the acetabulum measured 52.9% ± 16.6 on average. The femoral head was centred in 137 hips, eight hips showed a subluxation and there were no dislocations. In the ap view (available X-rays *n*=145), the femoral head shape was round in 33, elliptic in 65, aspheric in 26 and necrotic in 21 cases. In the false profile view (available X-rays *n*=127), the femoral head shape was round in 49, elliptic in 54, aspheric in 11 and necrotic in 13 cases. The orientation of the osteotomy was measured when a post-operative X-ray of adequate quality was available. The mean osteotomy angle was 11.2 ± 5.8 (−13 to 28). It was ideal in 69 cases, six osteotomies were too flat, five too steep, three intraarticular and one descending. The grade of OA increased significantly between surgery and follow-up. At follow-up, 53% of the patients had a Tönnis grade 3. Figure 3a–f depicts a representative case.

### Survival analysis results

**Fig. 4:**
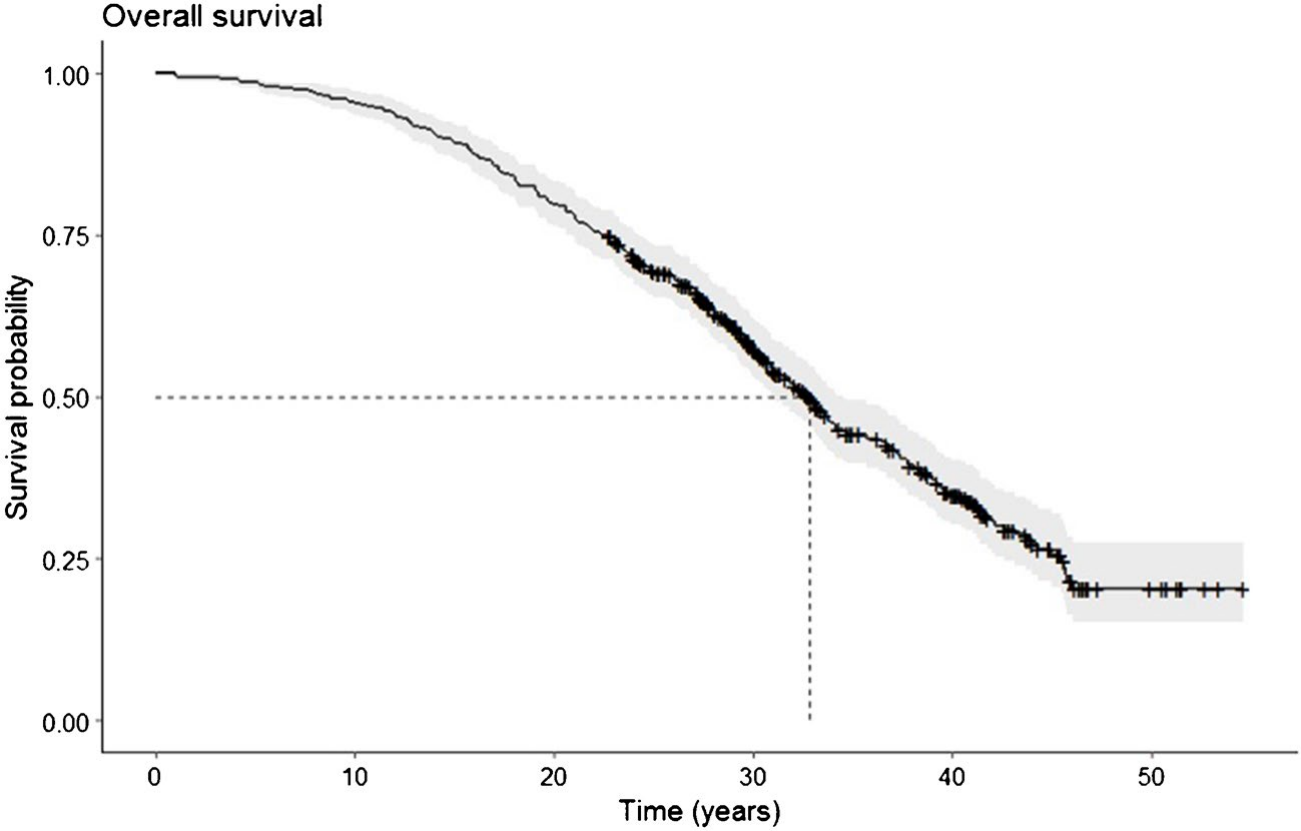
Kaplan–Meier survivorship curve for all hips indicating the probability of survival from the time of the CPO to the conversion to a THA

**Fig. 5:**
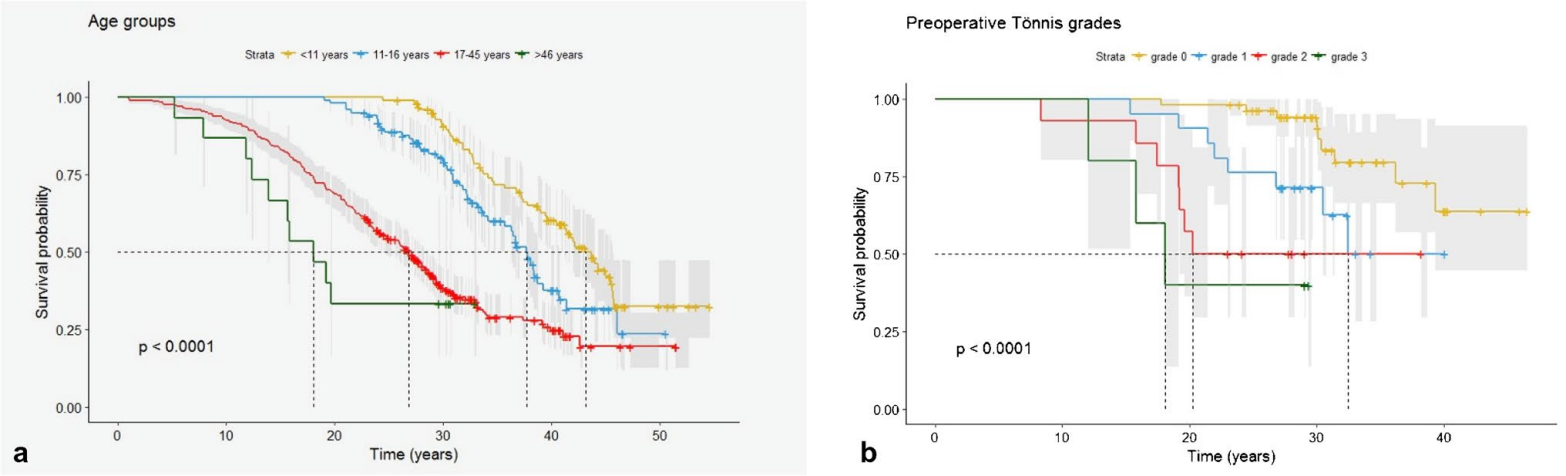
*p*-values refer to the log-tank test. **a** Kaplan–Meier estimates stratified for age groups. Younger patients had a significant better survivorship. **b** Kaplan–Meier estimates stratified for pre-operative Tönnis Grades. Lower grades of osteoarthritis had a significantly better survivorship

We calculated a cumulative survivorship of 79.8% (95% confidence interval (CI), 76.1–83.2%) (402 hips) at 20 years, 57.1% (95% CI, 52.8–61.8%) (217 hips) at 30 years and 35% (95% CI, 30.3–40.3%) (89 hips) at 40 years (Fig. 4 Overall Survival). The *p*-value refers to the log-rank test. Statistically significant differences were found for patients’ sex (*p* = 0.028), age groups (*p* < 0.0001) (Fig. 5a Overall Survival Stratified for Age), pre-operative diagnosis (*p* < 0.0001) and Tönnis OA grade (*p* < 0.0001) (Fig. 5b Overall Survival Stratified for Grade of Osteoarthritis). In summary, male patients, younger age, the pre-operative diagnosis of congenital dislocation or subluxation and a low grade of OA were positive prognostic factors. No differences were shown for previous conservative or surgical treatments. Clinical and radiological results are summarized in Table 3.

**Table 3:** Results

Kaplan–Meier survivorship rates stratified for different subgroups of patients				
Patient groups	**Kaplan–Meier survivorship rate, THA as endpoint (95% CI)**			
	20 years	30 years	40 years	
Overall survival	79,8% (76.1–83.2%)	57.1% (52.8–61.8%)	35% (30.3–40.3%)	
Sex				
Men	87.9% (79.9–96.7%)	72,2% (61–85.4%)	43% (27.8–65.9%)	
Women	78.3% (74.5–82.2%)	55.2% (50.6–60.2%)	33.4% (28.6–39.1%)	
Age				
<11 years	100%	90.6% (84.9–70.1%)	59% (49.6–70.1%)	
11 to 16 years	98% (95.2–100%)	79.3% (71.3–88.1%)	34.7% (24.2–49.9%)	
17 to 45 years	68.5% (63.4–74.1%)	37.8% (32.2–44.4%)	24.7% (18.8–32.4%)	
>46 years	33% 16.3–68.2%)	n.a.	n.a.	
Preoperative diagnosis				
Congenital hip dislocation	93.8% (85.7–100)	81.2% (68.8–96%)	55.8% (40.5–76.8%)	
Dyplastic subluxation	85.9% (77.8–94.9%)	69.5% (59–82%)	40.5% (29.6–55.5%)	
Hip dysplasia	83.3% (79.1–87.7%)	61.6% (56–67.8%)	36.3% (29.9–43.9%)	
Osteoarthritis	58.7% (50.2–68.7%)	30% (22.1–40.8%)	0%	
Tönnis OA grade				
Grade 0	97.2% (93.1–100%)	87.2% (76.8–99%)	63.8% (44.5–91.5%)	
Grade 1	85.7% (72–100%)	62.5% (42.9–91.1%)	n.a.	
Grade 2	50% (29.6–84.4%)	n.a.	n.a.	
Grade 3	40% (13–100%)	n.a.	n.a.	
**Radiographic measures**				
Radiographic measure	Preoperative	Postoperative	Follow-up	*P* value
L-CEA (°)	6.3 ± 13.0 (−30 to 31), *n*=94	45 ± 12.6 (20 to 79.7), *n*=89	43.6 ± 13.6 (10.9 to 78), *n*=151	+<0.0001, *0.0001, **<0.0001, ***NS
ACM (°)	61 ± 7.1 (23.5 to 73.1), *n*=80	56.9 ± 5.3 (42.3 to 71.4), *n*=75	53.1 ± 6.8 (37 to 74), *n*=148	+<0.0001, *0.0001, **<0.0001, ***<0.0001
AHI (%)	40.2 ± 14.1 (0 to 66.2), *n*=78	−8.1 ± 24.6 (−89.7 to 24.6), *n*=77	2.1 ± 16.6 (−45.5 to 47.3), *n*=149	<0.0001, *<0.0001, **<0.0001, ***NS
AI (°)	0.3 ± 0.2 (0 to 1.5), *n*=81	0.4 ± 0.3 (0.2 to 3.2), *n*=76	0.4 ± 0.1 (0.2 to 0.6), *n*=148	<0.0001, *0.04, **<0.0001, ***NS
A-CEA (°)	11.5 ± 23.2 (−23 to 42), *n*=14	46.9 ± 10.6 (30 to 63), *n*=8	42.6 ± 14.7 (−20 to 87.6), *n*=136	NS
Medialization (%)		52.9 ± 16.6 (22.7 to 100), *n*=82		
OT Angle (°)		11.2 ± 5.8 (−13 to 28), *n*=85		
Toennis Grade 0	55 (57.9%)	50 (54.9%)	0	
Toennis Grade 1	21 (22.1%)	21 (23.1%)	15 (9.8%)	
Toennis Grade 2	14 (14.7%)	14 (15.4%)	56 (36.8%)	
Toennis Grade 3	5 (5.3%)	6 (6.6%)	81 (53.3%)	
OA Toennis Grade	0.7 ± 0.9 (0.0 to 3.0), *n*=95	0.74 ± 0.94 (0.0 to 3.0), *n*=91	2.4 ± 0.67 (1.0 to 3.0), *n*=152	*0.32, **<0.0001, ***<0.0001

Survival probability in %, CI 95% in paretheses; *n.a*. not available

All values are given as mean and standard deviation, range in parentheses. n is the number of X-rays analysed for the individual measure and time point. *P* Values: + all time points (Friedmann test), *preoperative vs. postoperative, **preoperative vs. follow-up, ***postoperative vs. follow-up (Wilcoxon test for paired data). *NS* non significant. Normal values: L-CEA >25°, ACM <50°, AHI <30%, AI >0.34, A-CEA >25°, OT Ange 10°

## Discussion

To the best of our knowledge, this study is the longest follow-up with the largest number of patients treated with CPO in the literature. From our own institution, four case series were previously published which partly covered cases included in the present report [2, 14, 15, 26]. The publications of Chiari [2] and Windhager et al. [14] included mainly children with congenital dislocations or subluxation of their hips, while Lack et al. [26] reported on the results of patients with OA. In summary, the key findings were that an operation at a young age without signs of OA gave better clinical results and longer success. It was stated that the amounts of medial displacement and full coverage of the femoral head were the most important factors for good outcome. The current study finally confirmed these assumptions on the very long-term with adequate statistical power and evaluation by Kaplan–Meier survivorship analysis comparing different patients’ characteristics, which had been missing in the previously mentioned reports. The overall survivorship rate of 79.8% at 20 years, 57.1% at 30 years and 35% at 40 years confirmed this technique as a successful treatment of hip dysplasia and its different clincial presentations. There are a large number of international publications showing that the CPO was widely used and the gold standard until the 1980s when other techniques started to become more popular [27–34]. Just recently, Uchiyama et al. published their experience of valgus femoral osteotomy combined with CPO for OA of severely dysplastic hips with a survival rate of 46.9% after 20 years [35]. Apart from the originator’s institution’s reports [2, 14, 15, 26], Macnicol et al. published the largest series of CPO in developmental dysplasia of the hip (DDH) patients (191 patients/215 hips) with 94.7% survival at 20 and 85.5% survival at 30 years. Younger patients had better results [31]. Ito at al. presented a long-term follow-up of 163 patients (173 hips) with a mean follow-up time of 20 years (range 10 to 32.5). They compared a pre- or early OA group to an advanced OA group and found a significant lower survival at 30 years in the advanced OA group of 43.6% vs. 91.8% in the early OA group [33]. Ohashi et al. and Yanagimoto et al. also found better results in patients with lower OA grades. Femoral head deformities did not compromise the results [29, 32]. Despite of the advantages of reorientation osteotomies, which are the gold standard today, their long-term survival rates have not outperformed the ones of the CPO so far. Lerch et al. presented an excellent overview comparing survival rates of the Bernese PAO, rotational osteotomy, triple osteotomy and CPO and plotted it against the survivorship rates of THA in female patients younger than 50 years from the Swedish Hip Registry. All types of osteotomies showed a linear decline of survival up to 30 years. However, the same is true for THAs in young female patients. This longest follow-up of the PAO from its originator’s institution reported a 30-year survivorship of 43% with conversion to THA as an endpoint [12]. Younger patients without OA had the most benefit. Retroversion or post-operative over-coverage causing anterior impingement were factors associated with a poorer outcome [36, 37]. Van Stralen et al. showing a survival rate of 67% after 25 years in a series of 51 osteotomies in young adults, published the longest follow-up of triple osteotomies [11]. The authors also mention that THA after a triple osteotomy is notably easier than after a CPO. In this context, we confirmed recently that a prior CPO did not compromise the prerequisites for successful THA at a later stage and showed a survival of 93% after ten years [38]. In very young children, the Salter osteotomy showed superior results. The survival rate after open reduction and innominate osteotomy for late-presenting DDH at 30, 40 and 45 years was 99%, 86% and 54%, respectively [39]. The patient-reported outcome of our cohort was very positive reflected by the high rate of satisfaction and the positive results of the SF-36, despite of advanced OA and functional impairment. Patients’ expectations might have played a key role, which was also shown in the prospective ANCHOR cohort study of PAO patients [13].

Our study has limitations. It is a retrospective series. Pre-operative radiographs and clinical information were not available for all patients. Due to the long time span, only a third of the 1536 CPOs was available for follow-up. Of those 504 hips, 236 were physically examined and radiographed, while the other patients were interviewed on the telephone.

In conclusion, this study represents a very large series of CPOs with an ultra-long-term follow-up. Young ages, absence of OA and male sex were positive prognostic factors. The correction of dysplasia persisted over time, while the grade of OA increased significantly. Although the CPO is considered a salvage procedure nowadays, it achieved excellent long-term results even in indications, which would be treated differently today. The results can keep up with the ones of contemporary surgical techniques. We could demonstrate that the CPO stood the test of time in the surgical treatment of hip dysplasia.

## Data Availability

Original data are available with the corresponding author.
